# Safety and Feasibility of Extended Platelet‐Rich Fibrin as a Solo Barrier Membrane for Ridge Preservation: A Case Series

**DOI:** 10.1002/cre2.70282

**Published:** 2026-01-09

**Authors:** Nathan E. Estrin, Troy B. Tran, Alan Rene Espinoza, Paras Ahmad, Nima Farshidfar, Ryan Holmes, Yufeng Zhang, Richard J. Miron

**Affiliations:** ^1^ The University of Iowa College of Dentistry and Dental Clinics Iowa City Iowa USA; ^2^ Lake Erie College of Osteopathic Medicine School of Dental Medicine Bradenton Florida USA; ^3^ Private Practice, Global Dental Baja Mexicali Mexico; ^4^ Department of Research Advanced PRF Education Florida USA; ^5^ Department of Periodontology University of Bern Bern Switzerland; ^6^ Private Practice, True View Dental Radiology PLLC Boise Idaho USA; ^7^ Private Practice, Holmes Dental Radiology and Forensics PLLC Boise Idaho USA; ^8^ Department of Oral Implantology University of Wuhan Wuhan China

**Keywords:** albumin‐gel, albumin‐PRF, Bio‐PRF, extended‐PRF, horizontal centrifugation, leukocyte and platelet‐rich fibrin, l‐PRF

## Abstract

**Introduction:**

Platelet‐rich fibrin (PRF) has been commonly utilized for ridge preservation techniques either to introduce supraphysiological concentrations of autologous growth factors to the defect area (typically when mixed within a bone graft) or utilized alone as a solo “barrier” membrane. Noteworthy, however, one of the commonly reported drawbacks of PRF is its relatively short resorption period characterized by lasting roughly 2 weeks. This may therefore be insufficient for complete soft tissue closure and/or preventing soft tissue cells from infiltrating into the bony compartment. Recently, it was discovered that by heating plasma and denaturing albumin using the Bio‐Heat technology, the resorption properties of PRF could be extended from a standard 2–3 week period toward 4–6 months. The aim of the present human case series was to investigate for the first time the safety and applicability of utilizing this novel 100% autologous extended‐PRF (e‐PRF) membrane for ridge preservation.

**Materials and Methods:**

Twenty‐two patients requiring 22 single tooth posterior extractions were included in this case series. In all cases, atraumatic extractions were performed, and the sites were grafted using a combination of bone allograft and standard PRF to create “sticky bone.” Noteworthy, the barrier membrane utilized over top of the bone graft was the novel e‐PRF, which was utilized as a solo membrane in place of standard collagen or polytetrafluoroethylene (PTFE) membranes. Cone‐beam computed tomography scans were taken immediately after extractions and at 3 months postoperatively. Ridge width at 1, 3, and 5 mm apical to the crest, and buccal and lingual height dimensions were recorded at both time intervals. Additionally, buccal bone thickness at 1, 3, and 5 mm apical to the crest was recorded at baseline.

**Results:**

All extraction sites healed uneventfully without any postoperative complications. No clinical signs of infection or other complications were detected. The mean change in ridge width at 1, 3, and 5 mm apical to the crest was −1.27 ± 0.70, −0.94 ± 0.80, and −0.69 ± 0.79 mm, respectively. The mean change in buccal height and lingual height was −1.25 ± 1.16 and −0.94 ± 1.07 mm, respectively.

**Conclusions:**

The use of e‐PRF membranes in place of collagen membranes for ridge preservation was shown to be an effective, safe, and predictable treatment modality. The e‐PRF membranes can be fabricated at low cost with a barrier function that resorbs much more slowly over time when compared to standard PRF membranes. While this preliminary report demonstrated successful outcomes, additional randomized controlled clinical trials investigating soft tissue outcomes of the e‐PRF membranes when compared to more conventionally utilized membranes are further necessary to support these novel findings.

**Clinical Relevance:**

The use of e‐PRF membranes in ridge preservation is a safe, predictable, and all‐natural alternative to traditional membranes.

## 1 Introduction

Managing extraction sites is commonly the first step prior to successful dental implant placement to limit the dimensional alterations of hard and soft tissues following tooth extractions (Becker et al. 2016). Several clinical and animal studies have demonstrated that after dental extractions, almost two‐thirds of the dimensional loss that occurs happens within the first 6 months, and without proper ridge preservation techniques, the ideal position of dental implants may be compromised (Tan et al. 2012; Schropp et al. 2003; Van der Weijden et al. 2009). In an effort to reduce postextraction dimensional changes, ridge preservation is a commonly performed technique that utilizes a combination of bone grafts, membranes, and growth factors (Bassir et al. 2018). Platelet concentrates in particular have become more mainstream for socket grafting due to their regenerative potential, including their ability to promote faster revascularization of defect areas and the ability to recruit surrounding progenitor cells for accelerated healing (Miron et al. 2024). To achieve maximum ridge dimensions, it has been advised to mix platelet‐rich fibrin (PRF) in combination with a bone grafting material to create “sticky bone” for better handling properties in addition to adding autologous growth factors to the bony scaffold (Sohn et al. 2011; Yu et al. 2023; Feng et al. 2022).

In addition to using PRF to create “sticky bone,” PRF has also been utilized by clinicians as a solo grafting technique during extraction site management (Miron et al. 2021a). In a recent systematic review with meta‐analysis, the use of PRF on postextraction dimensional changes was investigated in 3 different comparisons: (1) PRF alone vs natural bone healing (12 studies), (2) PRF alone versus bone graft alone (4 studies), and (3) PRF alone vs PRF in combination with bone graft (3 studies) (Miron et al. 2021a). While PRF was able to limit dimensional changes postextraction when compared to natural bone healing, it was less effective when compared to a bone grafting material alone. One key study by Clark et al. evaluated four groups, including (1) natural wound healing, (2) PRF alone, (3) bone graft alone (freeze‐dried bone allograft; FDBA), and (4) FDBA combined with PRF (Clark et al. 2018). They concluded that the greatest loss of ridge height was noted in the natural blood clot group (3.8 ± 2.0 mm) when compared to the PRF group (1.8 ± 2.1 mm) and the FDBA group (2.2 ± 1.8) (Clark et al. 2018). The ridge was best maintained utilizing a combination of PRF + FDBA (only 1.0 ± 2.3 mm loss of ridge height), and the addition of PRF to FDBA also significantly favored greater new vital bone (Clark et al. 2018).

While more data are necessary to confirm potential advantages in bone healing when combining PRF with bone grafts, generally speaking, there is substantially more evidence supporting the use of PRF to aid in soft tissue wound healing when compared to hard tissues (Miron et al. 2017a, 2020, 2021a, 2017b; Amiri et al. 2023). Although PRF may be utilized as a “membrane” during ridge preservation to aid in faster wound closure, due to its rapid 2‐week resorption period, it has not been commonly selected over traditional barriers such as collagen resorbable membranes or polytetrafluoroethylene (PTFE) membranes owing to its fast degradation rate (Fujioka‐Kobayashi et al. 2021). This is due to the fact that if the membrane resorbs prior to complete wound closure, the grafted socket is left vulnerable with potential for bone grafting particle loss within the defect.

Owing to the fact that PRF alone could not be utilized as a barrier membrane, an interesting attempt was made in 2015 when Kawase and his colleagues introduced a heat‐compression technique with PRF membranes with the hypothesis that by heating PRF, it could improve the length of time required to degrade/resorb PRF (Kawase et al. 2015). It was observed in this breakthrough paper that the heat‐compression technique extended the degradation properties of PRF to greater than 3 weeks, significantly longer than the standard 2‐week PRF membranes (Kawase et al. 2015). This led to the hypothesis that heating platelet concentrates, specifically by denaturing proteins in the platelet‐poor plasma (PPP) layer rich in albumin, could favor a slower degradation rate and extend the lifespan of traditional PRF membranes.

Therefore, to overcome the quick degradation properties of platelet concentrates and better maintain volume stability, the albumin‐PRF (Alb‐PRF), also termed extended‐PRF (e‐PRF), protocol was developed (Fujioka‐Kobayashi et al. 2021; Gheno et al. 2021; Miron et al. 2024). Recent studies have successfully confirmed that PRF could be significantly extended from its standard 2‐week resorption time to greater than 4 months by heating the liquid PPP layer using a specialized medical device (Bio‐Heat technology) to denature albumin (Fujioka‐Kobayashi et al. 2021; Gheno et al. 2021; Miron et al. 2024). This heated version of plasma is being utilized in many areas of medicine and dentistry and has recently been the focus of extensive pre‐clinical and clinical research (Miron et al. 2024). Preclinical and clinical data has now found that this extended‐PRF can be utilized in orthopedic joint injections with superior outcomes, has been utilized for TMJ pain management more effectively, has been utilized to cover the lateral window during sinus grafting procedures and has been utilized as a substitute for collagen membranes and connective tissue grafts during recession coverage procedures (Miron et al. 2024; Estrin et al. 2024; Abdulhak et al. 2024).

Since one of the main limitations of PRP/PRF during extraction site coverage has historically been its short in vivo turnover rate, these extended‐PRF membranes were hypothesized as a potential replacement for collagen membranes or PTFE during ridge preservation techniques over standard bone allografts. The aim of this study was to showcase for the first time, through a consecutive case series, the safety and effectiveness of e‐PRF in ridge preservation as a solo barrier membrane.

## 2 Materials and Methods

### 2.1 Study Outline

**Figure 1:**
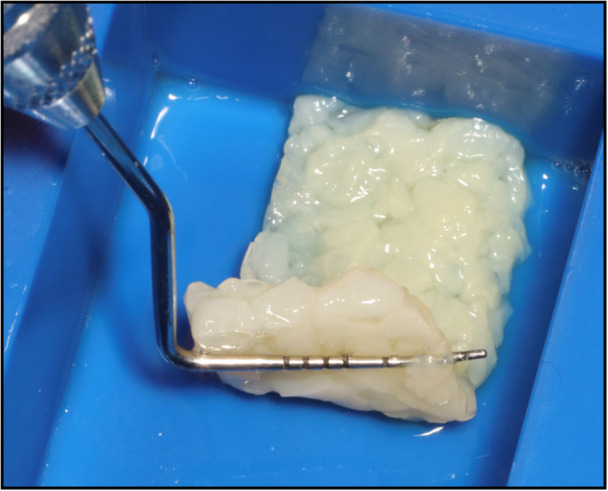
Clinical demonstration of the final extended‐PRF membrane ready to use for ridge preservation.

**Figure 2:**
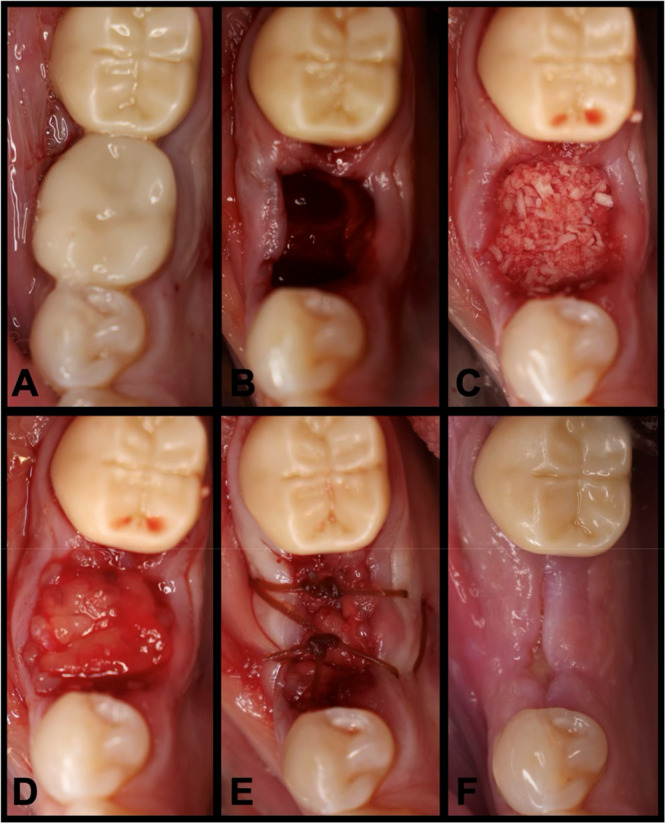
(A) Preoperative clinical photograph. (B) After extraction of tooth #30, the socket was degranulated with a spoon excavator leaving a socket with a buccal dehiscence present. (C) The extraction socket was grafted with “sticky bone.” (D) The e‐PRF membrane was then placed over the grafted socket and (E) sutured with 3‐0 chromic gut in mattress fashion. (F) The patient was brought back for a 2‐week postoperative evaluation. Note the excellent healing and near complete wound closure.

Before the start of data collection, an Institutional Review Board (IRB) exemption was obtained for the retrospective chart analysis from Sterling IRB (ID: 12243‐NEstrin). All identified patients had been treated by a single clinician (N.E.). 22 patients with 22 extraction sites were included in this case series. All patients were treated from 2023 to 2024 at Lakewood Ranch Dental, Sarasota, FL. All patients required the extraction of a hopeless posterior tooth and returned for implant placement. All patients who did not return for implant placement were omitted from this case series. Patients with uncontrolled diabetes or immunocompromised patients were also omitted from this study. See Table 1 for patient demographics and case distribution. Under local anesthesia, teeth were extracted with great care to preserve the surrounding bony walls with no flaps reflected. Thorough socket debridement was performed with curettes and carbide burs until hard osseous structure was reached. In all patients, the ridge preservation technique was completed utilizing “sticky bone” (described below) as the graft material and e‐PRF (described below; Figure 1). As all extractions were flapless, no effort was made to achieve primary closure, and the membranes were secured with 3‐0 chromic gut sutures. See Figure 2 for a case example at site #30. Patients were prescribed 500 mg of amoxicillin three times daily for 1 week and 800 mg of ibuprofen three times daily, as needed for pain. Patients were also placed on a homeopathic oral care recovery kit (StellaLife, StellaLife Inc., IL) for postoperative recovery (Estrin et al. 2022). All patients were seen for a postoperative check at 2 weeks and then again at 3 months for radiographic evaluation in preparation for implant placement.

**Table 1:** Distribution of clinical cases and patient demographics.

Age	Males/females	Molars/premolars	Maxilla/mandible	Intact sockets/buccal dehiscence
66.59 ± 9.76	15/7	16/6	9/13	15/7

### 2.2 Creating “Sticky Bone”

First, two 10 mL blue liquid‐PRF tubes and two 10 mL red solid‐PRF tubes were filled using a 21 G butterfly needle as per the manufacturer's recommendations (Bio‐PRF, Florida, USA). The tubes were all centrifuged together utilizing a horizontal centrifugation system at 700 RCF for 8 min (Bio‐PRF, Florida, USA). Following the spin cycle, the red top tubes produced a solid‐PRF clot whereas the blue top tubes remained liquid. To make “sticky bone,” the PRF clots from the red top tubes were removed, compressed in a PRF box for dehydration (Bio‐PRF), followed by transportation of the solid PRF membranes into a sterile bowl where it was subsequently minced/cut into small 1‐mm‐sized PRF fragments using sterile scissors. After the desired amount of cortical and cancellous allograft bone particulate was added (FDBA, MinerOss, BioHorizons) and mixed with the PRF fragments, the mixture was irrigated with liquid‐PRF drawn from the buffy coat zone of the blue liquid‐PRF tubes. Excess liquid‐PRF was drained with gauze and the allograft with PRF complex was left for 5 min to clot. A representative video describing the step‐by‐step procedure to accurately fabricate “sticky bone” is depicted in QR Code 1.


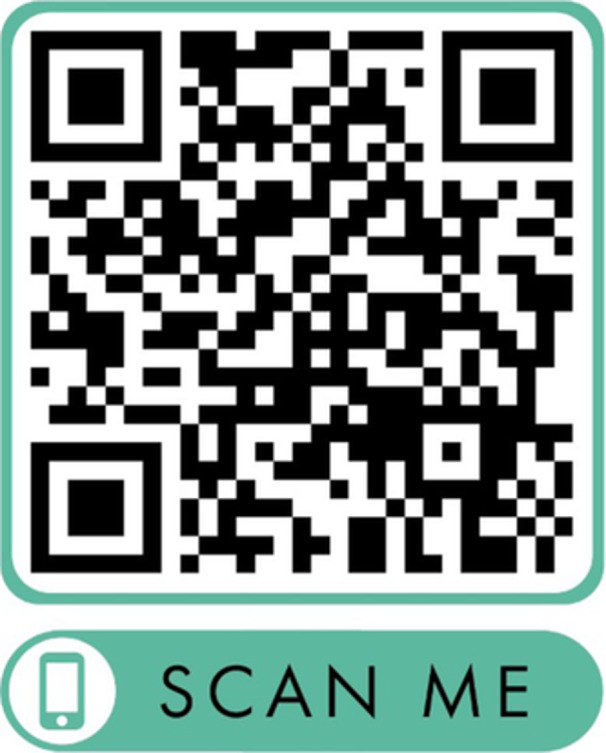


### 2.3 Creating the Extended‐PRF Membrane

To make the e‐PRF membrane, a standard protocol was followed according to those previously published (Fujioka‐Kobayashi et al. 2021; Gheno et al. 2021). Briefly, a 21‐G needle was utilized to draw two 10 mL liquid‐PRF tubes of blood from the patient and centrifuged at 700 RCF for 8 min utilizing a horizontal centrifuge (Bio‐PRF, Florida, USA). After centrifugation, one syringe was utilized to draw 2 mL of the PPP layer from one blue tube and placed in the Bio‐Heat Device (Bio‐PRF) for 10 min at a temperature of 75°C for serum albumin denaturation to produce the plasma gel with a 4–6 month working time. A second syringe was then utilized to draw 1–2 mL of liquid PRF from the buffy coat zone. The denatured albumin gel was then placed and condensed in the Bio‐Heat tray (Bio‐PRF) to the desired shape. The liquid PRF was then utilized to saturate the albumin gel in the Bio‐Heat tray and reintroduce the supraphysiological autologous growth factors into the matrix. The albumin gel and liquid‐PRF were then left for 10–15 min to set together (ideally in an incubator set to 37°C) before use. A representative video describing the step‐by‐step procedure to accurately fabricate the Alb‐PRF (also termed e‐PRF for extended‐PRF) is depicted in QR Code 2.


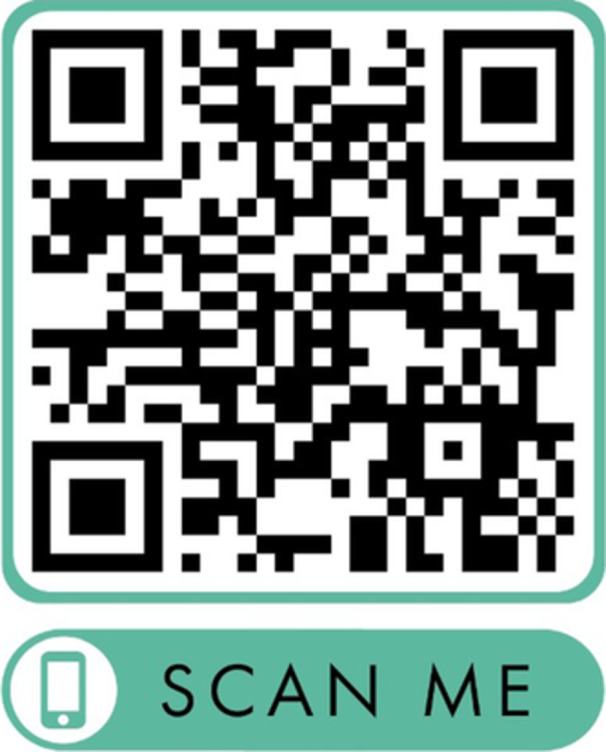


### 2.4 Radiographic Evaluation

**Figure 3:**
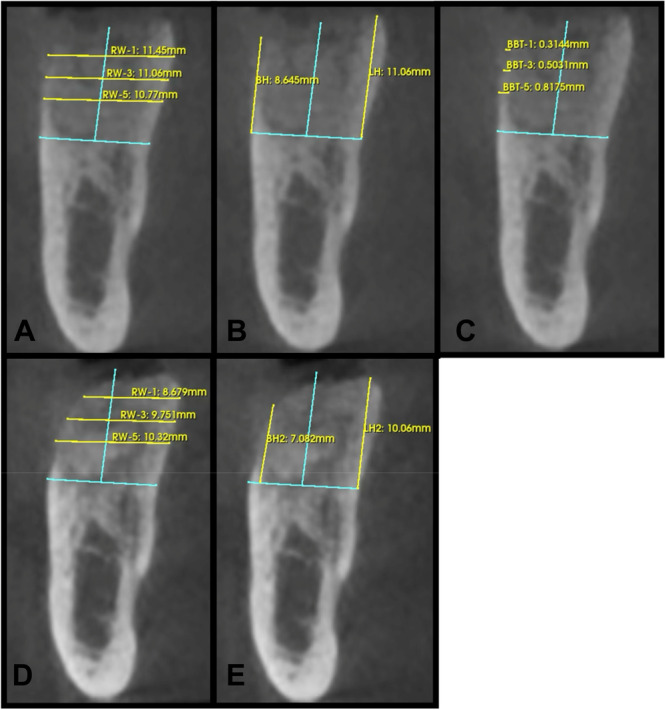
A clinical case of ridge preservation at site #30 demonstrating radiographic measurements. (A) Baseline measurements of ridge width at 1 mm (RW‐1), 3 mm (RW‐3), and 5 mm (RW‐5) apical to the crest. (B) Baseline measurements of Buccal height (BH) and Lingual Height (LH). (C) Baseline buccal bone thickness at 1 mm (BBT‐1), 3 mm (BBT‐3), and 5 mm (BBT‐5) apical to the crest. (D) 3‐month postoperative ridge width measurements at all levels and (E) 3‐month postoperative buccal and lingual height measurements. All measurements were taken with the same vertical and horizontal reference lines (in blue) at both time intervals.

For all patients a cone beam computed tomography (CBCT) scans (VAtech) were performed immediately after surgery (T0), and 3 months postsurgery (T1). The 3‐month postoperative scans were spatially matched to the immediate postoperative scans and positioned in the same coordinate system using an open‐source software package (Slicer version 5.8.1 www.slicer.org). The horizontal ridge width was measured on cross‐sectional images at 1 (RW‐1), 3 (RW‐3), and 5 mm (RW‐5) apical to the crest and perpendicular to a vertical reference drawn at the midpoint of the grafted socket. Buccal height (BH) and lingual height (LH) were measured on cross‐sectional images from the most apical portion of the socket to the most coronal portion of the ridge, parallel to the vertical reference line. Baseline buccal bone thickness was measured on cross‐sectional images at 1 mm (BBT‐1), 3 mm (BBT‐3), and 5 mm (BBT‐5) parallel to the horizontal reference line (Figure 3). All radiographic measurements and images were provided by Holmes Dental Radiology and Forensics, PLLC. The radiographic measurements were completed by the same oral and maxillofacial radiologist (R.H.). This case series has been reported in line with the PROCESS guideline (Agha et al. 2020).

## 3 Results

The primary goal of this case series was to evaluate the safety and effectiveness of the extended‐PRF membrane during ridge preservation. In this initial case series, 22 patients with 22 extraction sites were investigated. In total, 68.2% of the patients were male and 31.8% were female. The patient's ages ranged from 46 to 77 with an average age of 66.59 ± 9.76 years old. No patients were smokers. 40.9% of the extraction sites were located in the Maxilla and 59.1% were located in the mandible. 72.7% of extraction sites were molars and 27.3% of sites were premolars. 68.2% of the extraction sites were intact 4‐wall defects, and 31.8% of the sites were 3‐wall defects with a buccal dehiscence present. All sites were grafted utilizing “sticky bone” consisting of FDBA (MinerOss, BioHorizons, Alabama, USA) mixed with PRF. All sites utilized the novel 100% autologous e‐PRF membrane to cover the grafted site owing to its 4–6 month resorption period and were secured with 3‐0 chromic gut. No clinical signs of infection or other complications were detected. The clinical photographs in Figure 2 demonstrate a case utilizing the e‐PRF membrane with uneventful healing at site #30, and Figure 3 demonstrates an example of the radiographic measurements taken. The mean change in ridge width at 1 mm, 3 mm, and 5 mm apical to the crest was −1.27 ± 0.70, −0.94 ± 0.80, −0.69 ± 0.79 mm, respectively. The mean change in buccal height and lingual height was −1.25 ± 1.16 and −0.94 ± 1.07 mm, respectively. The average baseline buccal bone thickness at 1, 3, and 5 mm apical to the crest as recorded at −1.14 ± 0.81, −1.37 ± 0.90 , and −1.64 ± 1.06 mm, respectively. A summary of all radiographic measurements can be seen in Table 2.

**Table 2:** Summary of radiographic measurements at baseline (T0), and 3‐month follow‐up (T1).

	Baseline (mm)	3 months (mm)	Difference (mm)
RW‐1	12.15 ± 2.15	10.88 ± 2.38	−1.27 ± 0.70
RW‐3	12.68 ± 2.41	11.74 ± 2.67	−0.94 ± 0.80
RW‐5	12.84 ± 2.74	12.14 ± 2.72	−0.69 ± 0.79
BH	10.13 ± 1.42	8.88 ± 1.97	−1.25 ± 1.16
LH	10.90 ± 1.26	9.95 ± 1.61	−0.94 ± 1.07
BBT‐1	1.14 ± 0.81		
BBT‐3	1.37 ± 0.90		
BBT‐5	1.64 ± 1.06		

## 4 Discussion

Platelet‐rich fibrin therapy has been a valuable adjunct to ridge preservation surgeries and a mainstay in modern implant dentistry (Miron 2021). One of the major components of ridge preservation includes a barrier membrane in order to secure the bone graft particulate in place as well as prevent soft tissue ingrowth into the socket defect (Bassir et al. 2018). Traditional barrier membranes include both resorbable collagen membranes and non‐resorbable options such as PTFE (Bassir et al. 2018). While clinicians have reported utilizing standard PRF membranes, it is not a sufficient replacement over traditional membranes due to their quick degradation time despite contributing to more rapid soft tissue closure (Fujioka‐Kobayashi et al. 2021). By utilizing the Bio‐Heat technology to increase the degradation properties of PRF to upwards of 4 months Gheno et al. (2021) extended‐PRF provides an interesting opportunity for a novel 100% autologous membrane with the ability to release autologous growth factors that could be used as a potential long‐lasting alternative to traditional barrier membranes. The main objective of this case series was to evaluate for the very first time the safety and effectiveness of utilizing these novel e‐PRF membranes as a solo barrier for ridge preservation.

In this case series of 22 extraction sites, all sites healed uneventfully without postoperative infection or signs of inflammation. In our study, the average reductions in ridge width at 1, 3, and 5 mm apical to the crest were 1.27 ± 0.70, 0.94 ± 0.80, and 0.69 ± 0.79 mm, respectively. Despite these ridge reductions, residual ridge widths at 1 mm apical to the crest ranged from 6.90 to 15.1 mm with an average width of 10.88 ± 2.38 reflecting adequate bone dimensions for implant placement in all sites. Additionally, the average reduction in buccal and lingual ridge height was 1.25 ± 1.16 and 0.94 ± 1.07 mm, respectively. These findings are comparable to those reported by Clark et al. (2018) in which the ridge width reductions at coronal, middle, and apical levels were reported at 1.9 ± 1.1 mm, 1.7 ± 1.2 mm, and 1.6 ± 1.5 mm, respectively, in the FDBA + A‐PRF group. They also noted a 1.0 ± 2.3 mm reduction in ridge height measured at the mid‐buccal crest, which is slightly less than the height loss observed in our study. However, a direct comparison is limited by methodology differences since their study measured dimensional changes clinically with a stent while ours relied on radiographic assessment. Our results are also consistent with the findings of Thakkar et al. (2016) in which 0.75 ± 0.49 mm of horizontal bone loss at 180 days was observed following ridge preservation using demineralized freeze‐dried bone allograft (DFBDA) combined with PRF and secured with a collagen membrane. However, they also utilized clinical measurements versus our radiographic measurements. Direct comparisons to clinical studies with stent‐based caliper measurements must be interpreted with caution as our findings are based solely on radiographic assessment.

Although soft tissue outcomes were not assessed, the authors noted clinically favorable wound healing and rapid soft tissue coverage consistent with previous research on PRF in regenerative surgeries (Miron et al. 2017a). Nevertheless, additional randomized clinical trials are necessary with larger patient numbers to compare the healing of the e‐PRF membrane versus traditional solid‐PRF and collagen barrier membranes. Another advantage of PRF, which has been demonstrated in several studies, is that patients receiving PRF report significantly lower patient pain outcomes when compared to collagen membranes or other modalities (Meza‐Mauricio et al. 2021; Gülşen and Şentürk 2017; Ozgul et al. 2015; Lektemur Alpan and Torumtay Cin 2020; Bahammam 2018; Farshidfar et al. 2025c).

In addition to utilizing PRF as a barrier membrane for ridge preservation, the use of PRF in combination with bone grafting materials is becoming more utilized during standard ridge preservation (Miron 2021; Farshidfar et al. 2022a, 2022b). Specifically, by combining PRF with bone particulate, the fabrication of “sticky bone” allows for easier handling of the graft material complex with putty‐like consistency (Miron et al. 2024). The “sticky bone” consistency may further provide an advantage when grafting sockets in the maxilla exhibiting sinus communication by minimizing loose bone particles migrating into the maxillary sinus. However, none of the extraction sites in this case series exhibited a sinus communication postextraction.

A downfall of this study is a lack of comparator group with traditional membranes utilized in ridge preservation such as collagen or e‐PTFE. Regardless, the primary aim of this study was to demonstrate the safety and feasibility of e‐PRF membranes as an alternative to traditional barrier membranes for use in ridge preservation procedures, which was successfully demonstrated in this case series. Longitudinal and cross‐sectional studies with soft tissue outcomes of ridge dimensions and histological samples are now necessary to further investigate if the longer resorption time leads to higher vital bone formation and more favorable soft tissue parameters, especially when compared to traditional membranes. Another limitation of this study is that all radiographic measurements were performed by a single oral and maxillofacial radiologist. Future studies should incorporate multiple calibrated examiners and assess intra‐rater reliability to improve the validity of the radiographic outcomes.

Lastly, it remains crucially important to point out that the centrifugation protocols, tube types utilized, and medical device utilized for the production of PRF all matter significantly in order to maximize the regenerative properties of PRF (Farshidfar et al. 2025a, 2025b). In a recent study titled “Optimization of Platelet‐Rich Fibrin,” a series of key features were discussed to elevate clinical use of PRF (Miron et al. 2024). One important feature that has been discussed much more recently has been the effect of tube types on the final outcomes of PRF (Miron et al. 2024, 2022, 2021b; Wei et al. 2024). Many PRF tubes, though marketed as potentially being “chemical‐free,” are contaminated with additives such as silica or silicone, which may negatively impact the final production of PRF and patient healing (Miron et al. 2021b; Masuki et al. 2020; Tsujino et al. 2019a, 2019b).

## 5 Conclusion

The findings from the present first‐of‐its‐kind case series demonstrated that the extended‐PRF membranes could be utilized successfully and safely as an innovative outer barrier membrane during ridge preservation. This supraphysiological platelet concentrate greatly extends the resorption time of PRF from 2 weeks to 4–6 months, and as demonstrated in this case series, is a successful alternative to traditional collagen and PTFE membranes. Further randomized controlled clinical trials are now necessary comparing the extended‐PRF membranes with traditional barrier membranes on both ridge width maintenance post extraction as well as soft tissue closure rates and patient‐reported pain outcomes as these parameters seem to be improved with this novel technology.

## Author Contributions

**Nathan E. Estrin:** conceptualization, methodology, formal analysis, investigation, resources, writing – original draft preparation, writing – review and editing. **Troy B. Tran:** conceptualization, formal analysis, writing – review and editing. **Alan Rene Espinoza:** conceptualization, methodology, investigation, resources, writing – review and editing, project administration. **Paras Ahmad:** methodology, formal analysis, resources, writing – review and editing, supervision. **Nima Farshidfar:** conceptualization, methodology, formal analysis, investigation, resources, writing – review and editing. **Ryan Holmes:** methodology, formal analysis, resources, writing – review and editing. **Yufeng Zhang:** conceptualization, methodology, formal analysis, resources, writing – review and editing, supervision, project administration. **Richard J. Miron:** conceptualization, methodology, formal analysis, investigation, resources, writing – original draft preparation, writing – review and editing. All authors have read and agreed to the published version of the manuscript.

## Funding

The authors received no specific funding for this work.

## Consent

Informed consent was provided before the blood draw to conduct the outlined experiments.

## Conflicts of Interest

Richard J. Miron holds intellectual property on the production of PRF. All other authors declare that they have no conflicts of interest.

## Supporting information

12243‐NEstrin.

12243‐NEstrinNathan.

Exemption_or_Non‐Human.

ROCESS‐2025‐Checklist.

## Data Availability

Data available on request from the authors.
