# Supplementary material for: Safety and Feasibility of Extended Platelet‐Rich Fibrin as a Solo Barrier Membrane for Ridge Preservation: A Case Series
Source: Clin Exp Dent Res. 2026 Jan 9;12(1):e70282. doi: 10.1002/cre2.70282 (PMC12784283; doi:10.1002/cre2.70282)
Supplement: Supplementary file 1 — 12243‐NEstrin. [file CRE2-12-e70282-s003.pdf]

NOTICE OF APPROVAL OF WAIVER OF AUTHORIZATION

Date Issued:	August 26, 2024
Study Expiration Date:	Exempt
Principal Investigator:	Nathan Estrin, DMD, MS
Protocol:	Safety and Effectiveness of Extended Platelet Rich Fibrin as a Solo Membrane for Ridge Preservation: A Retrospective Case Series
IRB ID:	12243-NEstrin
Sponsor:	Nathan Estrin
Description of the PHI:	The PHI requested is the minimum amount of data needed to properly evaluate the success of the treatment and if there are any correlations with patient characteristic and success of procedure.

ACTION: Approval for Waiver of Authorization

Sterling IRB reviewed your request for waiver of authorization for your above captioned research project via **Exempt Review** and has found that your requested waiver of authorization, in the context submitted, meets the following criteria for approval:

1. The use or disclosure of protected health information involves no more than minimal risk to the individuals.

2. The alteration or waiver will not adversely affect the privacy rights and the welfare of the individuals.

3. The research could not practicably be conducted without the waiver of authorization.

4. The research could not practicably be conducted without access to and use of the protected health information.

5. The privacy risks to the individuals whose protected health information is to be used or disclosed is reasonable in relation to the anticipated benefits if any to the individuals, and the importance of the knowledge that may reasonably be expected to result from the research.

6. There is an adequate plan to protect the identifiers from improper use and disclosure.

7. There is an adequate plan to destroy the identifiers at the earliest opportunity consistent with the conduct of the research, unless there is a health or research justification for retaining the identifiers or such retention is otherwise required by law.

8. There are adequate written assurances that the protected health information will not be reused or redisclosed to any other person or entity, except as required by law, for the authorized oversight of the research study, or for other research for which the use or disclosure of protected health information would be permitted by regulation.

No alteration to the procedures described in the study protocol as reviewed on August 26, 2024 may be instituted unless this Board has reviewed and approved the continuation of the waiver of authorization.

Accounting rules apply to this waiver.

Sterling IRB reserves the right to report any violation of this approval to the Office for Civil Rights of the U.S. Department of Health and Human Services.

Kristi McGinnis
Signature Chairman or designee

Copy: Sponsor and/or CRO
