# Supplementary material for: Safety and Feasibility of Extended Platelet‐Rich Fibrin as a Solo Barrier Membrane for Ridge Preservation: A Case Series
Source: Clin Exp Dent Res. 2026 Jan 9;12(1):e70282. doi: 10.1002/cre2.70282 (PMC12784283; doi:10.1002/cre2.70282)
Supplement: Supplementary file 2 — 12243‐NEstrinNathan. [file CRE2-12-e70282-s002.pdf]

SterlingIRB
SAFEGUARDING YOUR RESEARCH

TYPE OF REVIEW – EXEMPTION FROM IRB REVIEW DETERMINATION

Determination Date:	August 26, 2024
IRB ID:	12243-NEstrin
Protocol:	Safety and Effectiveness of Extended Platelet Rich Fibrin as a Solo Membrane for Ridge Preservation: A Retrospective Case Series
Sponsor:	Nathan Estrin
Principal Investigator:	Nathan Estrin, DMD, MS

Sterling IRB is in receipt of submission materials for the above-referenced study.

Items Reviewed:
- Exemption or Non-Human Subjects Research Determination Request
- IRB Data collection for retrospective study. .xlsx (Data Collection Form)
- IRB epRF Socket grafting pilot study. 8:22:24 copy.docx (Protocol)

Based on the information available to the IRB, the Sterling IRB Chairman (or designee) has determined that:

The above-listed study is exempt from IRB review pursuant to the terms of the U.S. Department of Health and Human Service's Policy for Protection of Human Research Subjects at 45 C.F.R. §46.104(d).

Sterling IRB has determined that the following exemption category(ies) applies:
- Category 4 Exemption (DHHS)

Sterling IRB's exemption determination is based on the study-related information available to Sterling IRB as of the determination date listed above. Should any changes be made to the study subsequent to Sterling IRB's determination, this determination is no longer applicable. Examples of changes that would likely require IRB review:

- Removal of the consent process, or use of deception or incomplete disclosure.
- Significant changes to the recruitment procedures.
- Adding sensitive questions to a survey or interview process (e.g. questions regarding illegal activities; traumatic events such as childhood, sexual, or domestic abuse; suicide; or other probing questions that could reasonably place the subjects at risk of criminal or civil liability or be damaging to the subjects' financial standing, employability, educational advancement, or reputation).
- Collection of new or additional identifiable information.
- Changes to the data storage plan which may affect confidentiality.

As the project applicant you are responsible for following all policies of Sterling IRB as described in the Exemption or Non-Human Subjects Research Determination Request Submission Agreement which you accepted with project submission. It is your responsibility to ensure this project is conducted in accordance with applicable regulations (local, state and federal) as well as any requirements established by the IRB at the time of the review determination. Refer to the Investigator Handbook at www.sterlingirb.com for details of these responsibilities.

The Board will be apprised of this determination.
