# Supplementary material for: Safety and Feasibility of Extended Platelet‐Rich Fibrin as a Solo Barrier Membrane for Ridge Preservation: A Case Series
Source: Clin Exp Dent Res. 2026 Jan 9;12(1):e70282. doi: 10.1002/cre2.70282 (PMC12784283; doi:10.1002/cre2.70282)
Supplement: Supplementary file 3 — Exemption_or_Non‐Human. [file CRE2-12-e70282-s004.pdf]

SterlingIRB
SAFEGUARDING YOUR RESEARCH

View xForm - Exemption or Non-Human Subjects Research Determination Request

This form is completed to initiate the review of an exemption or non-human subjects research determination request. [APP756 Effective Date: 02.23.2024 Version: 3.6]

Exemption/NHSR data entry
- Submitted 08/25/2024 3:14 PM ET by Estrin, Nathan DMD, MS

General Information

Exemption or Non-Human Subjects Research Determination Request

This is an electronic smart form. You will be prompted to provide additional information based on how you answer questions throughout the form. Instructions and help notes are also included to assist you in providing required information. If you require further assistance anytime during the submission process, please contact us at 1-888-636-1062 to speak with a member of our Account Relations Department.

ATTENTION!
This form will automatically be deleted if it is NOT submitted within 90 days from initially opening the form.

Date this form was started:
08/23/2024

Date this form will be deleted if NOT submitted to Sterling IRB:
11/21/2024

Protocol Number: (***if applicable***)

No answer provided.

Protocol Title:

Safety and Effectiveness of Extended Platelet Rich Fibrin as a Solo Membrane for Ridge Preservation: A Retrospective Case Series

Sponsor:
NOTE: If there is no Sponsor, list the Principal Investigator's name.

Nathan Estrin

Which type of IRB determination are you requesting?

Exemption Determination Request

Please provide a brief description or attach a copy of the proposed protocol/project to explain the submission request:

No answer provided.

In needed, attach a copy of the proposed protocol/project here:

IRB epRF Socket grafting pilot study. 8:22:24 copy.docx	Protocol	08/23/2024 10:22 PM ET

Sites that will be submitted to Sterling IRB are located in: *(check all that apply)*

United States

Research Site Location (US):

Research Site Name:
Lakewood Ranch Dental

Address, line 1:
6270 Lake Osprey Drive

Address, line 2:
No answer provided.

City:
Sarasota

State:
Florida

ZIP Code:
34240

If there are more than one research site location, you may attach a list here that includes the Research Site Name and full Address for each location:
No answer provided.

Click "Next" below to continue to the next page in this form. Click "Save for Later" to save your progress and return later.

Project Contacts

Contact Information

Who will be the Principal Investigator (PI) for this project?

In the box below, enter the email address of the PI for this project.
Please be sure to check the spelling of the information entered.

Estrin, Nathan DMD, MS
Email: drnathan@estrinperiodontics.com

NOTE: If the Form displays "Contact not found", follow the instructions at the bottom of this page to create a profile for the Contact. Once the profile is created, you may return to this page and re-enter their email address in table above.

For this project, is the PI the primary contact person for communications to/from the IRB?
Yes

PI's Phone Number/Extension:
4124915408

Is the PI also the billing contact person for this project?
Yes

Other than the PI and primary contact person above, are there any additional contact persons for this project?

No

Follow these steps to add each person whose email address was not found as a new Contact in the system:

1. Click the "*New SilverLink Contact Form*" link below to add each new Contact (1 person per Form). The *New SilverLink Contact Form* will open in a separate browser tab.

2. Complete and submit the New SilverLink Contact Form. This will create a profile for the Contact. You will receive a confirmation email once the Contact has been added. This process typically take 1 to 2 minutes. You may continue completing this Form while your request is processing.

3. This process may be repeated to add additional Contacts, if needed.

4. When you receive the confirmation email,you may return to this page of this Form and enter the email address of the newly-added Contact in the appropriate box above.

User had the option to start a different form here.

NOTE: You will need to complete a separate *New SilverLink Contact Form* for each person whose email address was not found. Once a person is available as a Contact in the system, they will not need to be added again for future submissions.

Click "Previous" to go back one page in this form. Click "Next" to continue to the next page in this form.
Click "Save for Later" to save your progress and return later.

Billing Information

Billing Information

Billing Contact Person:

You indicated that the Billing Contact is the same as the PI for this study.

Billing Preferences:

How do you wish to receive invoices?
Email

PO Number: ***(if applicable)***
No answer provided.

Special Billing Instructions:
No answer provided.

Click "Previous" to go back one page in this form. Click "Next" to continue to the next page in this form.
Click "Save for Later" to save your progress and return later.

General Protocol/Project Information

Has this protocol/project previously been reviewed and disallowed by another IRB?

No

Is the site under the jurisdiction of a local IRB (i.e., the site requires that IRB oversight of this study be waived or authorized to Sterling IRB)?

NOTE: If Yes, the IRB Jurisdiction Form must be completed with this submission. The IRB Jurisdiction Form will appear later in this smart form if required.

No

Will the proposed protocol/project include direct contact with participants?

NOTE: If Yes, a copy of the proposed data collection form, questionnaire or interview questions to be used, and the proposed consent form or script to be used must be included with this submission.

No

Will the proposed protocol/project include collection of data from records?

NOTE: If Yes, a copy of the proposed data collection form to be used must be included with this submission.

Yes

Please attach a copy of of the proposed data collection form to be used here:

IRB Data collection for retrospective study.xlsx	Data Collection Form	08/25/2024 3:04 PM ET

Will the proposed protocol/project include collection of Protected Health Information (PHI)?

Yes

Please verify which of the following is being included with this submission:
Application for Waiver or Alteration of Authorization

Click "Previous" to go back one page in this form. Click "Next" to continue to the next page in this form.
Click "Save for Later" to save your progress and return later.

Application for Waiver or Alteration of Authorization

Application for Waiver or Alteration of Authorization

This form is to request a waiver or alteration of authorization for use/disclosure of Protected Health Information.

General Information

Sponsor:
No answer provided.

Protocol Title:
Safety and Effectiveness of Extended Platelet Rich Fibrin as a Solo Membrane for Ridge Preservation: A Retrospective Case Series

Protocol No.:
No answer provided.

Principal Investigator:
Nathan Estrin

Describe the reason that you are asking for this waiver or alteration and why the research could not practicably be conducted without the waiver or alteration of authorization:

We are requesting this waiver because this study will be a retrospective study in which data collection will occur without interaction with patients. These patients have already received treatment.

Please explain how the use or disclosure of protected health information (PHI) involves no more than minimal risk to the privacy of the individuals:

Most of the information is basic and without the patient's chart (which only the PI will have access to), it will not be possible to identify the patient based on the data provided.

Explain why the research could not practicably be conducted without access to and use of the PHI:

The goal of this retrospective study is to evaluate the safety and success of a novel membrane. Data from the patients chart including implant success, tooth number, and any complications will need to be recorded.

Describe your plan to destroy the identifiers (identifiers must be destroyed at the earliest opportunity unless there is a health or research justification for retaining the identifiers or such retention is otherwise required by law). If there is no intent to destroy the identifiers, please justify your retention of this information:

The patient's will be assigned a number on the excel sheet during data collection. After completion of the retrospective study the excel document in which links the patient's number to their name will be deleted.

Describe your plan to protect the identifiers from improper use and disclosure:

On the excel document that will be shared among other investigators, only the patient's number will be reported and not their name. It will not be possible to identify the patient's name based on the data collected. Only the PI will have access to patient's charts.

Please describe the PHI that will be accessed, used or disclosed in the course of the proposed research and explain why the PHI requested is the minimum necessary to achieve the goals of this research.
NOTE: Under federal regulations, investigators may obtain only the minimum necessary PHI to achieve the goals of the research.

The PHI requested is the minimum amount of data needed to properly evaluate the success of the treatment and if there are any correlations with patient characteristic and success of procedure.

Who will have access to this information?

Principal Investigator

Are they required to sign confidentiality agreements?
Yes

In what form will this information be maintained?

Electronic

Does the database have a secure network, with limited access and password protection?
Yes

Investigator's Assurance

I provide assurance that:

1. *Only the minimum necessary PHI for the purposes of this research will be used or disclosed.*
2. *Protected health information obtained in this study will not be reused or disclosed to any other person or entity other than those authorized to receive it, except: a) as required by law, b) for authorized oversight of the research, or c) in connection with other research for which the use or disclosure of that PHI is permitted by the HIPAA Privacy Rule.*

Attestation:

By submitting this form, I attest that the information provided in this application is true and accurate and is submitted by, or under the authority of, the Principal Investigator who agrees to comply with the above.

Person Completing this Form:

Estrin, Nathan DMD, MS

Click "Previous" to go back one page in this form. Click "Next" to continue to the next page in this form.
Click "Save for Later" to save your progress and return later.

Funding Information

The source of funding for this protocol/project will be: ***(check all that apply)***

NOTE: If the research is federally funded, a complete copy of the grant application and/or federal contract or rationale why this documentation is unavailable must be provided with this submission.
*In addition, a completed copy of the **Sterling IRB Authorization Agreement** (available at **http://www.sterlingirb.com/forms/** under "**Forms for Institutions**") must also be included with this submission.*

Other (description required)

Please identify other funding source(s):
No funding provided

Click "Previous" to go back one page in this form. Click "Next" to continue to the next page in this form.
Click "Save for Later" to save your progress and return later.

(US) Is project subject to DHHS regulation?

If No to either of the following questions, the project is not human subjects research under DHHS regulations. If the project is subject to FDA and/or DHHS regulation and you would like to request an exemption determination, complete the exemption section that outputs based on the responses that were provided.

Does this project meet either of the following definitions of human subjects?

Yes

Definition of Human Subject:
DHHS: 45 CFR 46.102(e)(1). "Human subject" means a living individual about whom an investigator (whether professional or student) conducting research: (i) Obtains information or biospecimens through intervention or interaction with the individual, and uses, studies, or analyzes the information or biospecimens; or (ii) Obtains, uses, studies, analyzes, or generates identifiable private information or identifiable biospecimens. Intervention includes both physical procedures by which information or biospecimens are gathered (e.g., venipuncture) and manipulations of the subject or the subject's environment that are performed for research purposes. Interaction includes communication or interpersonal contact between investigator and subject. Private information includes information about behavior that occurs in a context in which an individual can reasonably expect that no observation or recording is taking place, and information that has been provided for specific purposes by an individual and that the individual can reasonably expect will not be made public (e.g., a medical record). Identifiable private information is private information for which the identity of the subject is or may readily be ascertained by the investigator or associated with the information. An identifiable biospecimen is a biospecimen for which the identity of the subject is or may readily be ascertained by the investigator or associated with the biospecimen.

Does this project meet the following definition of research?

Yes

Definition of Research:

DHHS: 45 CFR 46.102(l). "Research" means a systematic investigation, including research development, testing and evaluation, designed to develop or contribute to generalizable knowledge. Activities that meet this definition constitute research for purposes of this policy, whether or not they are conducted or supported under a program which is considered research for other purposes. For example, some demonstration and service programs may include research activities.

The following activities are deemed not to be research: (1) Scholarly and journalistic activities (e.g., oral history, journalism, biography, literary criticism, legal research, and historical scholarship), including the collection and use of information, that focus directly on the specific individuals about whom the information is collected; (2) Public health surveillance activities, including the collection and testing of information or biospecimens, conducted, supported, requested, ordered, required, or authorized by a public health authority. Such activities are limited to those necessary to allow a public health authority to identify, monitor, assess, or investigate potential public health signals, onsets of disease outbreaks, or conditions of public health importance (including trends, signals, risk factors, patterns in diseases, or increases in injuries from using consumer products). Such activities include those associated with providing timely situational awareness and priority setting during the course of an event or crisis that threatens public health (including natural or man-made disasters); (3) Collection and analysis of information, biospecimens, or records by or for a criminal justice agency for activities authorized by law or court order solely for criminal justice or criminal investigative purposes; and (4) Authorized operational activities (as determined by each agency) in support of intelligence, homeland security, defense, or other national security missions.

Sterling IRB defines a "systematic investigation" as an activity designed to test a hypothesis and to permit conclusions that develop or contribute to generalizable knowledge to be drawn; includes a research plan that sets forth an objective and a set of procedures to reach that objective.

Sterling IRB defines "generalizable knowledge" as information expressed in theories, principles, and statements of relationships that can be widely applied (i.e. both to the population being studied and possibly to other populations).

Click "Previous" to go back one page in this form. Click "Next" to continue to the next page in this form.
Click "Save for Later" to save your progress and return later.

(US) Is project subject to FDA regulation?

If No to either of the following questions, the project is not human subjects research under FDA regulations. After completing this section, please proceed to the next Section to determine whether the project is subject to DHHS regulation.

Does this project meet either of the following definitions of human subjects?

No

Definition of Human Subject:

- *FDA: 21 CFR 50.3(g), 56.102(e). **"Human subject"** means an individual who is or becomes a participant in research, either as a recipient of the test article or as a control. A subject may be either a healthy human or a patient.*
- *FDA: 21 CFR 812.3(p). "**Subject**" means a human who participates in an investigation, either as an individual on whom or on whose specimen an investigational device is used or as a control. A subject may be in normal health or may have a medical condition or disease. For medical device studies involving in vitro diagnostics and unidentified tissue specimens, the FDA defines the unidentified tissue specimens as human subjects.*

Does this project meet either of the following definitions of research?

No

Definition of Research:

- *FDA: 21 CFR 50.3(c), 56.102 (c). "**Clinical investigation**" means any experiment that involves a test article and one or more human subjects and that either is subject to requirements for prior submission to the Food and Drug Administration under section 505(i) or 520(g) of the act, or is not subject to requirements for prior submission to the Food and Drug Administration under these sections of the act, but the results of which are intended to be submitted later to, or held for inspection by, the Food and Drug Administration as part of an application for a research or marketing permit. The term does not include experiments that are subject to the provisions of part 58 of this chapter, regarding nonclinical laboratory studies. The terms research, clinical research, clinical study, study, and clinical investigation are deemed to be synonymous for purposes of part 56.*
- *FDA: 21 CFR 812.3 (h). "**Investigation**" means a clinical investigation or research involving one or more subjects to determine the safety or effectiveness of a device.*

NOTE: Since you answered "No" to one or more of the above statements, *your submission is not human subjects research under FDA regulations.*

Click "Previous" to go back one page in this form. Click "Next" to continue to the next page in this form.
Click "Save for Later" to save your progress and return later.

(US) DHHS Exemption Categories

U.S. DHHS Exemption Categories at 45 CFR 46.104(d):

(1) Research, conducted in established or commonly accepted educational settings, that specifically involves normal educational practices that are not likely to adversely impact students' opportunity to learn required educational content or the assessment of educators who provide instruction. This includes most research on regular and special education instructional strategies, and research on the effectiveness of or the comparison among instructional techniques, curricula, or classroom management methods.

(2) Research that only includes interactions involving educational tests (cognitive, diagnostic, aptitude, achievement), survey procedures, interview procedures, or observation of public behavior (including visual or auditory recording) if at least one of the following criteria is met:

(i) The information obtained is recorded by the investigator in such a manner that the identity of the human subjects cannot readily be ascertained, directly or through identifiers linked to the subjects;
(ii) Any disclosure of the human subjects' responses outside the research would not reasonably place the subjects at risk of criminal or civil liability or be damaging to the subjects' financial standing, employability, educational advancement, or reputation; or
(iii) The information obtained is recorded by the investigator in such a manner that the identity of the human subjects can readily be ascertained, directly or through identifiers linked to the subjects, and an IRB conducts a limited IRB review to make the determination required by §46.111(a)(7).

NOTE: *This exemption does not apply to research with children except for research that meets criteria a. or b. and involves only educational tests or observations of public behavior when the investigator(s) do not participate in the activities being observed.*

(3) Research involving benign behavioral interventions in conjunction with the collection of information from an adult subject through verbal or written responses (including data entry) or audiovisual recording if the subject prospectively agrees to the intervention and information collection and at least one of the following criteria is met:

(A) The information obtained is recorded by the investigator in such a manner that the identity of the human subjects cannot readily be ascertained, directly or through identifiers linked to the subjects;
(B) Any disclosure of the human subjects' responses outside the research would not reasonably place the subjects at risk of criminal or civil liability or be damaging to the subjects' financial standing, employability, educational advancement, or reputation; or
(C) The information obtained is recorded by the investigator in such a manner that the identity of the human subjects can readily be ascertained, directly or through identifiers linked to the subjects, and an IRB conducts a limited IRB review to make the determination required by §46.111(a)(7).

Benign behavioral interventions are brief in duration, harmless, painless, not physically invasive, not likely to have a significant adverse lasting impact on the subjects, and the investigator has no reason to think the subjects will find the interventions offensive or embarrassing. Provided all such criteria are met, examples of such benign behavioral interventions would include having the subjects play an online game, having them solve puzzles under various noise conditions, or having them decide how to allocate a nominal amount of received cash between themselves and someone else.

If the research involves deceiving the subjects regarding the nature or purposes of the research, this exemption is not applicable unless the subject authorizes the deception through a prospective agreement to participate in research in circumstances in which the subject is informed that he or she will be unaware of or misled regarding the nature or purposes of the research.

(4) Secondary research for which consent is not required: Secondary research uses of identifiable private information or identifiable biospecimens, if at least one of the following criteria is met:
(i) The identifiable private information or identifiable biospecimens are publicly available;
(ii) Information, which may include information about biospecimens, is recorded by the investigator in such a manner that the identity of the human subjects cannot readily be ascertained directly or through identifiers linked to the subjects, the investigator does not contact the subjects, and the investigator will not re-identify subjects;
(iii) The research involves only information collection and analysis involving the investigator's use of identifiable

health information when that use is regulated under 45 CFR parts 160 and 164, subparts A and E, for the purposes of "health care operations" or "research" as those terms are defined at 45 CFR 164.501 or for "public health activities and purposes" as described under 45 CFR 164.512(b); or
(iv) The research is conducted by, or on behalf of, a Federal department or agency using government-generated or government-collected information obtained for nonresearch activities, if the research generates identifiable private information that is or will be maintained on information technology that is subject to and in compliance with section 208(b) of the E-Government Act of 2002, 44 U.S.C. 3501 note, if all of the identifiable private information collected, used, or generated as part of the activity will be maintained in systems of records subject to the Privacy Act of 1974, 5 U.S.C. 552a, and, if applicable, the information used in the research was collected subject to the Paperwork Reduction Act of 1995, 44 U.S.C. 3501 et seq.

(5) Research and demonstration projects that are conducted or supported by a Federal department or agency, or otherwise subject to the approval of department or agency heads (or the approval of the heads of bureaus or other subordinate agencies that have been delegated authority to conduct the research and demonstration projects), and that are designed to study, evaluate, improve, or otherwise examine public benefit or service programs, including procedures for obtaining benefits or services under those programs, possible changes in or alternatives to those programs or procedures, or possible changes in methods or levels of payment for benefits or services under those programs. Such projects include, but are not limited to, internal studies by Federal employees, and studies under contracts or consulting arrangements, cooperative agreements, or grants. Exempt projects also include waivers of otherwise mandatory requirements using authorities such as sections 1115 and 1115A of the Social Security Act, as amended.
Each Federal department or agency conducting or supporting the research and demonstration projects must establish, on a publicly accessible Federal Web site or in such other manner as the department or agency head may determine, a list of the research and demonstration projects that the Federal department or agency conducts or supports under this provision. The research or demonstration project must be published on this list prior to commencing the research involving human subjects.

(6) Taste and food quality evaluations and consumer acceptance studies,
(i) if wholesome foods without additives are consumed, or
(ii) if a food is consumed that contains a food ingredient at or below the level and for a use found to be safe, or agricultural chemical or environmental contaminant at or below the level found to be safe, by the Food and Drug Administration or approved by the Environmental Protection Agency or the Food Safety and Inspection Service of the U.S. Department of Agriculture.

(7) Storage or maintenance for secondary research for which broad consent is required: Storage or maintenance of identifiable private information or identifiable biospecimens for potential secondary research use if an IRB conducts a limited IRB review and makes the determinations required by §46.111(a)(8).
NOTE: *In order for the research to qualify for this exemption category, please attach a copy of the broad consent.*

(8) Secondary research for which broad consent is required: Research involving the use of identifiable private information or identifiable biospecimens for secondary research use, if the following criteria are met:

(i) Broad consent for the storage, maintenance, and secondary research use of the identifiable private information or identifiable biospecimens was obtained in accordance with §46.116(a)(1) through (4), (a)(6), and (d);
(ii) Documentation of informed consent or waiver of documentation of consent was obtained in accordance with §46.117;
(iii) An IRB conducts a limited IRB review and makes the determination required by §46.111(a)(7) and makes the determination that the research to be conducted is within the scope of the broad consent referenced in paragraph (d)(8)(i) of this section; and
(iv) The investigator does not include returning individual research results to subjects as part of the study plan. This provision does not prevent an investigator from abiding by any legal requirements to return individual research results.

NOTE: *In order for the research to qualify for this exemption category, please attach a copy of the broad consent.*

Does this protocol/project meet one or more of the exemption categories listed above?

Yes

Please check *all* exemption categories that apply for this protocol/project. *At least* one category must be selected:
Category 4 Exemption (DHHS)

Click "Previous" to go back one page in this form. Click "Next" to continue to the next page in this form.
Click "Save for Later" to save your progress and return later.

Submission Agreement

SUBMISSION AGREEMENT

By submitting this form, I attest that the information provided in this application is true and accurate and is submitted by, or under the authority of, the Principal Investigator and/or Sponsor/CRO listed herein who agrees that this protocol/project will be conducted in an ethical manner and that the following ethical standards will be met:

- *The research involves no more than minimal risk to participants.*
- *The research does not involve prisoners as research participants except for research aimed at involving a broader subject population that only incidentally includes prisoners.*
- *If participants will be enrolled, selection is equitable.*
- *If there is recording of identifiable information, there are adequate provisions to maintain the confidentiality of the data.*
- *If there are interactions with participants, there will be a consent process that will disclose such information as:*
  - *That the activity involves research*
  - *A description of the procedures*
  - *That participation is voluntary*
  - *Name and contact information for the Researcher*
- *There are adequate provisions to maintain the privacy interests of subjects*

Check here to confirm the above

IRB ID #:

12243

NOTE: The IRB ID # for your protocol/project submission is listed above. Please include this IRB ID # on all future correspondence pertaining to this submission.

Person who started this Form:

Estrin, Nathan DMD, MS

Email: drnathan@estrinperiodontics.com

Person who submitted this Form:

*NOTE: The identity of the user who clicks "**Submit**" on the next page will automatically be captured once the xForm has been submitted to the IRB.*

Estrin, Nathan DMD, MS

Email: drnathan@estrinperiodontics.com

This is the last page of this form.

Click "Previous" to go back one page. Click "Save for Later" to save your progress and return later.
Click "Next" to proceed to the submission page.

On the next page you can click "Save for Later" to save your progress, or "Submit" to submit this form to Sterling IRB for review.

Copyright ©2000-2024 Tech Software. All Rights Reserved.
2024.7.7718.0/Release/5a24929 | GCWAWS1 | 2024-08-26 18:31:33Z

Powered By IRBManager
